# A qualitative examination of human papillomavirus vaccine access, beliefs, and behaviors among adult males aged 18–35: applying the Health Stigma and Discrimination Framework

**DOI:** 10.3389/fpubh.2026.1751794

**Published:** 2026-06-05

**Authors:** Jacquelin I. Cordero, Louis D. Brown, Jesús M. Alemán, María E. Fernández, Serena A. Rodríguez, Eva M. Moya

**Affiliations:** 1 Department of Health Promotion and Behavioral Sciences, School of Public Health, University of Texas Health Science Center at Houston, Houston, TX, United States; 2 Border Biomedical Research Center, University of Texas at El Paso, El Paso, TX, United States; 3 Center for Health Promotion and Prevention Research, School of Public Health, University of Texas Health Science Center at Houston, Houston, TX, United States; 4 Interdisciplinary Health Sciences PhD Program, College of Health Sciences, University of Texas at El Paso, El Paso, TX, United States; 5 Institute for Implementation Science, University of Texas Health Science Center at Houston, Houston, TX, United States; 6 Department of Social Work, College of Health Sciences, University of Texas at El Paso, El Paso, TX, United States

**Keywords:** Critical Discourse Analysis (CDA), Health Stigma and Discrimination Framework (HSDF), human papillomavirus (HPV) vaccination behaviors, intersecting stigmas, male health, modifiable cancer risk factors, multi-level determinants, sexual and reproductive health

## Abstract

**Background:**

Males, who disproportionately account for 83% of oropharyngeal cancer cases, are underrepresented in health promotion research, including human papillomavirus (HPV) vaccination studies. The initial exclusion of males in HPV vaccine research, policies, and interventions has negatively affected male vaccine uptake. Furthermore, stigma, a well-documented barrier to healthcare engagement, has been overlooked in HPV vaccination behaviors. This study aimed to provide a more contextualized understanding of HPV vaccine disparities among adult males by applying the Health Stigma and Discrimination Framework (HSDF) to examine how stigma is produced, enacted, and sustained across social systems, which may impact male vaccine uptake.

**Methods:**

This qualitative study analyzed previously collected data from virtual group interviews conducted in 2021 with a convenience sample of 13 vaccine-eligible males aged 18–35 residing in El Paso, Texas, United States. Participants completed a demographic survey and participated in semi-structured discussions about their knowledge, attitudes, and experiences with HPV and vaccination. Data were analyzed by employing the Critical Discourse Analysis (CDA) method and the Health Stigma and Discrimination Framework (HSDF) to guide the methodological and analytical processes, helping to map stigma processes and mechanisms across individual, interpersonal, and structural levels to identify multi-level influences on male HPV vaccine behavior.

**Results:**

Analysis identified four overarching themes reflecting the discursive construction of stigma processes and mechanisms, spanning proximal to distal levels of influence. At the individual level, (1) *male sexual health and manhood* reflects how internalized sex-specific beliefs and masculine norms fostered shame and discomfort around male sexual health, often framing males as unaffected by HPV rather than vulnerable or in need of vaccination. At the interpersonal level, (2) *cultural and familial forces* captures how discourses of intergenerational silence, religious morality, and sexual health taboos perpetuated stigma processes and mechanisms, constraining open discussion about HPV and vaccination. At the systemic level, (3) *structural barriers to vaccination* reflects how policy environments, institutional practices, and health system norms perpetuate systemic-level obstacles restricting vaccine access. Finally, at the multi-level, (4) *stigma resistance* reflects counter-narratives that highlight affirming, peer-informed, perspectives and reframed male vaccination as legitimate and necessary.

**Discussion:**

Findings provide an in-depth examination of how stigma processes and mechanisms—rooted in masculine norms, sexual health taboos, intergenerational silence, misinformation, and institutional exclusion—shape HPV vaccination access, beliefs, and behaviors among vaccine-eligible adult males. In contrast, participants expressed resistance to these stigmatizing narratives by reframing male vaccination as a necessary and achievable health behavior when supported by peer-informed, culturally resonant messaging. These findings highlight the need for multi-level interventions that replace stigmatizing discourses and practices with affirming male narratives in HPV and broader sexual and reproductive health prevention efforts.

## 1 Introduction

Human papillomavirus (HPV) is the most prevalent sexually transmitted infection (STI), affecting around 79 million people in the United States (U.S.), with an additional 14 million people contracting the virus yearly (1). Persistent infection with oncogenic HPV types causes the majority of cervical, vaginal, vulvar, anal, oropharyngeal, and penile cancers, contributing to approximately 48,000 new HPV-associated cancers annually in the U.S. (1–3).

Oropharyngeal cancers (OPCs) are the most common HPV-associated cancer site, representing 45% of all HPV-related cancers. OPCs disproportionally impact males, with approximately 83% of OPC cases occurring in adult males (4). Unlike routine screenings for cervical and anal cancer, OPCs lack a standard/routine screening which leads to later-stage diagnoses requiring more aggressive treatments (5–8). Additionally, males in the U.S. are less likely to receive the HPV vaccine during adolescence or adulthood thus increasing their likelihood of acquiring and transmitting oncogenic HPV types, and putting them and their sexual partners at a higher risk of developing HPV-related cancers (9, 10).

HPV acquisition, transmission, and the development of HPV-associated cancers are associated with modifiable risk factors that can be addressed through preventative measures, including HPV vaccination, routine screening, and prompt treatment of precancerous lesions (11, 12). Initially approved for females in 2006, HPV vaccination expanded to include males in 2011 and adults aged 27–45 years in 2019 (10, 13–15). In the U.S., HPV vaccines are covered by health insurance under the Affordable Care Act (ACA) guided by national immunization guidelines set forth by ACIP (16).

Despite expanded eligibility, adult HPV vaccination coverage remains low, particularly among males (13, 17). National survey data (2018–2022) showed that only 47% of adults aged 18–26 years (females: 61%; males: 39%) and 16% of adults aged 27–45 years (females: 77%; males: 23%) have received at least one dose, with males persistently underrepresented compared to females (18, 19). These persistent disparities highlight the need to better understand the facilitators and barriers of HPV vaccination among vaccine-eligible U.S. males, particularly following the 2019 age-expansion.

Stigma is a well-documented barrier to health-seeking behavior, engagement in care, and treatment adherence across a range of health conditions (20, 21). Stigma refers to socially constructed negative beliefs that lead to labeling, stereotyping, separation, and discrimination within contexts of unequal power (22). Individuals are marked (stigmatized) through social labeling, judgment, or distinction when they are perceived as deviating from dominant norms related to identity (e.g., sex, age, and ethnicity) or health-related behaviors and statuses (e.g., STI/HPV and vaccination) (20–22).

The Health Stigma and Discrimination Framework (HSDF) conceptualizes stigma as a multi-level social process organized into the domains of drivers, facilitators, manifestations, outcomes, impacts, and resistance (21). The HSDF also accounts for how these social processes may intersect and compound, creating intersecting stigmas. Among males, a cultural tension exists between dominant norms that frame males as disinterested in sexual and reproductive health-protective or preventative behaviors and the health systems that fail to meaningfully engage or retain adult male patients (23, 24). This tension manifests through intersecting forms of stigma experienced by males across multiple levels of influence (20, 21). One example is seen in adult male HPV vaccination behaviors, such as those in underserved and international border-region populations (20, 21, 25).

Underserved and predominantly racial/ethnic minority populations often experience a disproportionate burden of cancer-related health disparities and remain largely underrepresented in cancer prevention research (26). For example, the U.S.–Mexico border region, including El Paso, Texas, offers a unique context to examine these dynamics due to its binational, bicultural population and longstanding health disparities (26, 27). A study using national survey data (2019 and 2022) of adult HPV vaccination rates (aged 18–26 years) showed that foreign-born Hispanics had the second lowest vaccination prevalence and were 24% less likely to be vaccinated compared to their US-born counterparts (28). Notably, a formative cross-sectional survey questionnaire study among a convenience sample of adults exploring HPV knowledge, attitudes, and behaviors in the El Paso region revealed that perceptions of health-related community stigma were associated with HPV vaccine hesitancy among vaccine-eligible adults (aged 18–45 years) (29). Building on these findings, a qualitative approach allows for deeper exploration of the local drivers and dimensions of intersecting stigmas that shape individual and subsequent population-level HPV vaccine outcomes in unique contexts, such as El Paso (20, 30).

This qualitative study aimed to provide a more contextualized understanding of HPV vaccine disparities among adult males. We apply the Health Stigma and Discrimination Framework (HSDF), guided by the Social Ecological Model (SEM), to examine how stigma is produced, enacted, and sustained across social systems (21). Our study mapped these stigma processes across individual, interpersonal, and structural levels to identify multi-level influences on male HPV vaccine behavior.

## 2 Materials and methods

### 2.1 Qualitative paradigm and methodological orientation

This study was grounded in a critical constructivist paradigm, informed by critical theory and action research principles that emphasize social justice and ethical engagement (31, 32). A critical constructivist approach acknowledges that knowledge and realities are socially and historically constructed, shaped by power relations that privilege certain perspectives while marginalizing others (31). Researchers in this tradition interrogate not only the processes that legitimize knowledge but also those that silence or exclude alternative voices (31, 32). Consistent with this paradigm and the study's aim to explore intersecting stigmas, qualitative inquiry was selected as the most appropriate methodological approach (20). To enhance transparency and rigor, this study followed the Standards for Reporting Qualitative Research (SRQR) guidelines in its design and reporting (33).

### 2.2 Context

#### 2.2.1 Geographical situation

El Paso County, Texas (population 840,758), includes four cities (El Paso, Horizon, San Elizario, and Socorro), two towns (Anthony and Clint), one village (Vinton), and over 362 identified *colonias* (unincorporated settlements) (27). El Paso is the largest Texas community along the U.S.-Mexico border (34).

Since 1994, the Health Resources and Services Administration (HRSA) has designated most areas in El Paso County as both Medically Underserved Areas (MUAs) and Health Professional Shortage Areas (HPSAs) (35). Hispanics make up 83% of the population, followed by white people/person(s) at 12%, and African Americans at 4%. Roughly 25% of the population is foreign born, and 71% of residents speak another language at home (36). Individuals aged 18 to 64 years make up approximately 53% of the population, 30% of whom lack healthcare access (36). Approximately 75% of the population holds a high school diploma (or equivalent), and 23% have obtained a bachelor's degree or higher (36).

#### 2.2.2 Parent study

In line with a critical constructivist paradigm, the parent study was collaboratively developed with research team members from the El Paso region who identified both as males and females within the HPV vaccine–eligible age group (31). The lead researcher of the current study (J.I.C) co-led the development of the data collection tools and participated in data collection of the parent study. The purpose of the parent study was to better understand male perspectives about HPV and the HPV vaccine in order to inform an adult vaccine promotion intervention (24). During March 9, 2021–June 5, 2021, participants were recruited electronically, using multi-media flyers, from partner community-based organizations, private and public agencies, and federally qualified health centers (out-reach) as well as the research team's local [El Paso] social circles (in-reach). Participant eligibility criteria included: identifying as male; aged 18–35 years; living in the El Paso area; and able to use electronic or mobile communication devices. Participants were deemed ineligible and excluded if they participated in previous parent project studies or could not participate in electronic data collection processes. Based on institutional COVID-19 policies at the time of data collection, in-person research activities were not available.

Eligible participants were invited to join the study. Once consented, participants completed a 20-item survey to capture demographics and health-related information. The survey was delivered electronically via the QuestionPro^®^ platform. Participants were then assigned to group interviews in a manner that reflected both logistical considerations and participant preference: (1) recruitment order, (2) preferred language (English, Spanish, or bilingual), and (3) availability. There were 13 participants (*N* = 13), organized into three group interview sessions. Due to limited study resources and in-person research activities during COVID-19, data saturation was not sought nor feasible.

The semi-structured bilingual interviews (English/Spanish) were audio- and video-recorded via Zoom. The semi-structured interview guide based on the *ecosocial perspective*—a systems lens to study complex interrelationships between inequitable exposure to risk factors and adverse disease outcomes—was co-developed by academic researchers who specialize in male health within the priority age group (37–39). The parent study researchers drew insight from previous qualitative studies conducted as part of the parent study (40, 41). The interview guide enabled the researchers to explore participants lived experiences through two main sections: (1) understanding of and thoughts about HPV, and (2) potential impacts of COVID-19 on receiving information about HPV and related needs in the El Paso area.

Video and audio recorded data were de-identified, transcribed, translated, and back-translated (Spanish and bilingual groups) between April and June 2021 by trained researchers with similar ethnic and cultural backgrounds as the study participants. The University of Texas at El Paso's Institutional Review Board approved the recruitment and participation of parent study subjects (IRB No. 1441487).

### 2.3 Measures

The current study utilized all participant (*N* = 13) parent study data. The survey questionnaire included three sections: (1) demographics, (2) healthcare access and utilization, and (3) sexual and reproductive health history. Demographic variables included age (in years), race (White person(s)/people, Hispanic, African American, multiracial, and other), ethnicity (Hispanic and non-Hispanic), level of education (no formal education to terminal degree), employment status (yes, no, and other), and occupation (open answer response). Interview question themes included (1) salient knowledge, attitudes, and behaviors associated with HPV, (2) messaging on HPV vaccine, (3) cancer prevention and screening, and (4) prospective HPV education campaign delivery methods.

### 2.4 Data processing

All data were password-protected, stored, and retrieved from the parent study institution's OneDrive platform. Quantitative survey data was analyzed using SPSS (v. 29.0) (42). Descriptive statistic frequencies were reported for all continuous (mean and standard deviation) and categorical (levels, frequencies, and percentages) variables. Prior to qualitative analysis, all transcripts were verified for accuracy and regional translations following transcription (co-authors J.M.A. and J.I.C.). Qualitative interview data were analyzed using Dedoose Version 10.0.25 (43).

### 2.5 Analytic approach and framework

In line with the critical constructivist paradigm, and best practices to address cancer-related health disparities, the qualitative analysis was collaboratively conducted (J.I.C.) with a Hispanic male health researcher within the HPV vaccine eligible age group, who was raised, educated, and resides in the El Paso region, (J.M.A.) (31, 44). Complementing the paradigm, our study employed the Critical Discourse Analysis (CDA) method and the Health Stigma and Discrimination Framework (HSDF) to guide the methodological and analytical processes (21, 45). Specifically, CDA enabled us to explore, thematically and discursively, how intersecting social identities, ideologies, and power dynamics (e.g., social, political, and historical contexts) are represented and negotiated in discourses about HPV, such as the subtle ways in which stigma is communicated (expressed) and internalized (experienced) among vaccine-eligible males (45). Additionally, HSDF guided our study and facilitated a deeper analysis, framing, and interpretation of multiple and intersecting forms of stigma, contextualizing how stigma is produced, sustained, and resisted across individual, interpersonal, and structural levels of influence (21, 46). HSDF helped emphasize the broader social, cultural, political, and economic forces that shape stigma, by recognizing that everyone is susceptible to both experiencing and expressing (practicing) stigma within the systems in which we function; while recognizing the potential of individuals and systems to act as agents of change (21).

### 2.6 Data analysis

The research dyad (J.M.A. and J.I.C.) used the CDA approach and HSDF framework to collaboratively develop the coding scheme by translating these approaches into themes, domains, and individual codes. Applying the CDA approach, we analyzed various forms of text (i.e., written, spoken, and visual communication) to uncover underlying patterns, ideologies, and social representations (i.e., experienced and expressed stigma) (45). We accomplished this through inductively incorporating affective preliminary codes (i.e., emotion and value coding) and elemental methods (i.e., descriptive and *in vivo* coding) to interpret underlying meanings in the data (45, 47). We used the HSDF framework to identify and interpret stigma-related processes across individual, interpersonal, and structural levels deductively using the HSDF domains: *drivers and facilitators* (negative and positive), *intersecting stigmas, manifestations* (experiences and practices), *outcomes* (affected populations), and *impacts* (long-term consequences) (21, 48).

Codes were defined in Dedoose, a qualitative analysis program, and agreed upon prior to coding. Both researchers (J.M.A. and J.I.C.) independently coded an initial transcript and then met to discuss and refine the application of the scheme; consensus was reached before coding the remaining transcripts. This coding scheme was then iteratively refined through thematic coding of the transcripts, identifying patterns and constructing overarching themes situated within individual, interpersonal, and systemic levels of influence by the researchers. Disagreements were resolved through reflexive dialogue, drawing on both researchers' distinct perspectives to reach consensus.

### 2.7 Positionality and reflexivity

The analysis was informed by the complementary positionalities of the research dyad (J.M.A. and J.I.C.). Both researchers are bilingual (English/Spanish) and deeply rooted in the El Paso, Texas region, where they live and work. These shared linguistic and cultural ties to the community enhanced their ability to engage with the data in a culturally responsive and authentic manner.

The lead researcher and female coder (J.I.C.) approached the data through a critical, intersectional-informed lens, grounded in social justice and a commitment to engaging males as partners in the research process. Her academic training is grounded in social work and public health, with a concentration in U.S.–Mexico border health issues, social justice, and the capacity building of historically marginalized communities (49). J.I.C. was raised, educated, and resides in the El Paso region, where she has worked in the field of HPV cancer prevention since 2018 (40, 50, 51). The male coder (J.M.A.), a member of the HPV vaccine–eligible male community, brought an insider perspective on masculine norms, cultural narratives, and lived experiences in the border region. These positionalities shaped how the team engaged in reflexive dialogue and ensured that findings reflected the complexity of participants' experiences while honoring the ethos of conducting research *with*, not *on*, the priority population (31, 32).

## 3 Results

### 3.1 Descriptive data

Descriptive characteristics of the participants (*N* = 13) are summarized in Table 1. The mean participant age was 25.69 years (SD = 5.87). The majority (92.31%) self-identified as Hispanic, and over half (53.85%) identified as White person(s)/people. Majority of participants (53.85%) reported a university (4-year) degree or higher, and 61.54% reported their employment status as “other” (e.g., student and gig work). More than half (53.85%) participants reported being single, followed by those in monogamous relationships (30.77%), dating (7.69%), or identifying their status as “other” (7.69%).

**Table 1:** Descriptive characteristics.

Characteristics of male group interview participants (***N*** = 13)	
Variable	*n* (*%*)
Level of education	
High school	4 (30.77%)
Technical school	1 (7.69%)
College (2 years)	1 (7.69%)
University (4 years)	5 (38.46%)
Masters	1 (7.69%)
Doctorate	1 (7.69%)
Race	
White	7 (53.85%)
Other/Hispanic	6 (46.15%)
Ethnicity	
Non-Hispanic	1 (7.69%)
Hispanic	12 (92.31%)
Employment status	
No	5 (38.46%)
Other	8 (61.54%)
Relationship status	
Single	7 (53.85%)
Dating	1 (7.69%)
Monogamous relationship	4 (30.77%)
Other	1 (7.69%)
Where do you usually get vaccinated? (Select all that apply)	
Clinic	8 (61.54%)
Doctors office	3 (23.08%)
Nurse practitioner	1 (7.69%)
15.6-7.4,-14.3242ptPharmacy	1 (7.69%)
Where do you receive information regarding [adult] vaccines? (Select all that apply)	
Clinic	7 (31.82%)
Doctors office	4 (18.18%)
Family member/Friend/Neighbor	1 (4.55%)
Nurse practitioner	2 (9.09%)
Internet	6 (27.27%)
	*M* (SD)
Age	25.69 (5.87)

Questions with “select all that apply” may ≠ 13.

### 3.2 Healthcare access, utilization, and sexual health service scales

Healthcare access, utilization, and sexual health service scale items and descriptive statistics are presented in Table 2. Among the participants (*N* = 13), approximately half (53.85%) reported having had at least one dose of the HPV vaccine. Fewer than half (38.46%) reported having any type of health coverage; however, the majority (84.6%) reported seeing a healthcare provider within the past year. Additionally, more than two-thirds (69.23%) reported having received sexual or reproductive health services in their lifetime. Nearly all participants (92.31%) had engaged in sexual activity at some point in their lifetime, and approximately half of the participants (61.54%) reported being currently sexually active and using some form of sexual health protection.

**Table 2:** Descriptive statistics.

Healthcare access, utilization, and sexual health history scales (*N* = 13)	
Variable	*n* (*%*)
HPV vaccine status	
None	2 (15.38%)
1 Dose	4 (30.77%)
2 Dose	2 (15.38%)
3 Dose	1 (7.69%)
Not Sure	4 (30.77%)
Health insurance	
Yes	5 (38.46%)
No	7 (53.85%)
Type of insurance	
Medicare	1 (7.69%)
Private insurance plan	4 (30.77%)
N/A	8 (61.54%)
Most recent healthcare provider visit	
Less than a month ago	5 (38.46%)
1–3 months ago	3 (23.08%)
6–12 months ago	3 (23.08%)
Over a year ago	2 (15.38%)
Ever received sexual or reproductive health services	
Yes	9 (69.23%)
No	4 (30.77%)
Ever engaged in sexual activity	
Yes	12 (92.31%)
No	1 (7.69%)
Currently sexually active	
Yes	8 (61.54%)
No	5 (38.46%)
Currently using protection	
Yes^*^	8 (61.54%)
No	5 (38.46%)
^*^If Yes Type of protection used (Select all that apply)	
Birth control pills	1 (6.67%)
Male condoms	8 (53.33%)
Pull out method	1 (6.67%)
Other/(N/A)	5 (33.33%)

Questions with “select all that apply” may ≠ 13. The asterisk (^*^) indicates a conditional follow-up item. Participants who answered “Yes” to the preceding question regarding current use of protection were subsequently asked the follow-up question: *If Yes, Type of protection used (Select all that apply).

### 3.3 Critical discourse analysis

**Figure 1:**
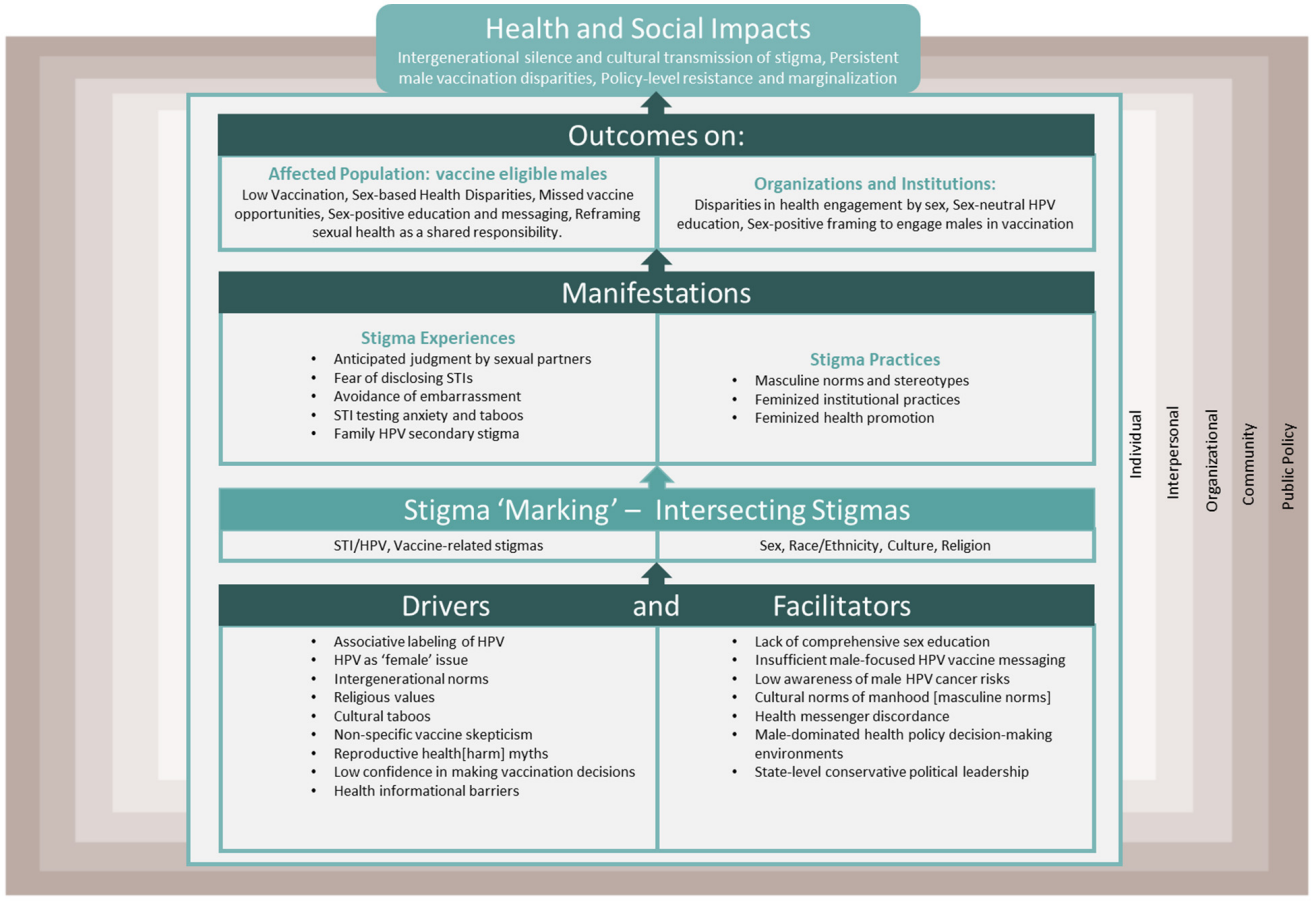
Thematic map illustrating embedded themes and participant codes organized within Health Stigma and Discrimination Framework (HSDF) domains. Adapted from (21), licensed under CC BY 4.0.

Thematic coding of the data, guided by the Critical Discourse Analysis (CDA) approach and the Health Stigma and Discrimination Framework (HSDF) identified four overarching themes reflecting the discursive construction of stigma process and mechanisms, spanning proximal to distal levels of influence. The domains, along with their embedded themes and codes organized within the HSDF framework, are summarized in Table 3 and visually depicted in Figure 1. The figure illustrates how intersecting stigmas are produced through layered influences at individual, interpersonal, and systemic levels, with themes embedded within the framework's domains (stigma processes and mechanisms) rather than presented as discrete categories.

**Table 3:** Summary of HSDF domains, overarching themes, and derived participant codes.

HSDF Domains	Domain description	Theme	Theme description	Code
Stigma drivers and facilitators	Reflect the underlying social, cultural, and institutional mechanisms that shape how males engage with sexual health and HPV prevention; influence awareness, comfort, and decision-making and often operate at multiple levels.	1. Male Sexual Health and Manhood: Individual level	Explores how manhood and sex-specific health beliefs shape males' engagement with HPV cancer prevention.	Sexual Health Stigma and Associative Labeling
				Manhood and Masculine Norms
		2. Cultural and Familial Forces: Intrapersonal level	Examines how intergenerational silence, religious values, and cultural taboos surrounding sex perpetuate the misinformation and stigma associated with HPV and its vaccination.	Cultural and Familial Silence around Sexual Health
				Misinformation and Fear about Vaccines
		3. Structural Barriers to Vaccination: Systemic Level	Explores how policy environments, institutional practices, and health system norms create structural barriers to HPV vaccination.	Institutional Practices, Policies, and Decision-Maker Influence
Stigma marking	Refers to the ways individuals are socially labeled, judged, or distinguished due to perceived deviations from dominant social norms related to identity or health behaviors/health status; form stigma processes and mechanisms that shape marginalization.	4. Multi-level: Stigma Resistance	Expressed views that challenged [resisted] dominant norms.	Positive Deviance
Stigma manifestations	Reflect how stigma is experienced or expressed in relation to HPV and its vaccination; this includes behaviors, language, or attitudes reflecting internalized fear, social discomfort, and perceived judgment.	1. Male Sexual Health and Manhood: Individual Level	Explores how manhood and sex-specific health beliefs shape males' engagement with HPV cancer prevention.	Stigma Experiences
				Stigma Practices
Stigma outcomes	Describe the direct and measurable effects of stigma on health behaviors, healthcare engagement, and public health reach; these mechanisms represent the consequences of stigma as it materializes within healthcare systems and policy environments.	3. Structural Barriers to Vaccination: Systemic Level	Explores how policy environments, institutional practices, and health system norms create structural barriers to HPV vaccination.	Delayed or Avoided Vaccination and Sexual Health Services
				Self-Sacrificing Health Behaviors Rooted in Provider/Protector Identity
Stigma impacts	Reflect long-term structural consequences, shaping health behaviors and access to care across individual, familial, and institutional levels; reinforce inequities and discourage individuals from seeking preventive care.	2. Cultural and Familial Forces: Intrapersonal Level	Examines how intergenerational silence, religious values, and cultural taboos surrounding sex perpetuate the misinformation and stigma associated with HPV and its vaccination.	Intergenerational Silence and Cultural Transmission

The final overarching themes aligned stigma processes and mechanisms within ecological levels of influence to identify multi-level intervention opportunities (21, 46). At the individual level, (1) *male sexual health and manhood* reflects how internalized sex-specific beliefs and masculine norms fostered shame and discomfort around male sexual health, often framing males as unaffected by HPV rather than vulnerable or in need of vaccination. At the interpersonal level, (2) *cultural and familial forces* captures how discourses of intergenerational silence, religious morality, and sexual health taboos perpetuated stigma processes and mechanisms, constraining open discussion about HPV and vaccination. At the systemic level, (3) *structural barriers to vaccination* reflects how policy environments, institutional practices, and health system norms perpetuate systemic-level obstacles restricting vaccine access. Finally, at the multi-level (4) *stigma resistance* reflects counter-narratives to challenge dominant narratives, articulating affirming, peer-informed perspectives that reframed male vaccination as legitimate and necessary.

#### 3.3.1 Individual level

##### 3.3.1.1 Male sexual health and manhood

This theme reflects how sex-specific health beliefs and discourses of masculine norms fostered shame and discomfort around male sexual health, often framing males as unaffected by HPV rather than vulnerable or in need of vaccination. In line with CDA, the research dyad framed this theme as *manhood*, rather than *masculinity*, mirroring participants' own language and centering their sense of identity, dignity, and social recognition, while acknowledging that manhood is shaped by broader societal expectations.

Several codes contributed to this theme. For example, the code **sexual health stigma and associative labeling** describes participants' unfamiliarity discussing HPV, as well as stigma processes and mechanisms linked to male sexual health.

Participants described their perceptions and associations of HPV:

“*It kind of harkens the idea of a sexually transmitted disease… sounds like HIV but with a P” or “[just another] three-lettered disease…”*

Participants also described how social taboos surrounding STI testing stigmatize male engagement in sexual health:

“*I don't see any challenges for men, other than maybe being taboo and people being, I guess embarrassed of having to go and be tested for [STIs].”*

Another code, **manhood and masculine norms**, highlights how cultural ideals—such as *machismo*, emotional stoicism, and expectations to appear unaffected by health risks— discourages help-seeking and undermines male HPV vaccine uptake.

Participants articulated how culturally specific norms, particularly among Hispanic males along the U.S.–Mexico border, discourages help-seeking:

“*… specific challenges for men… masculinities or gender norms or you know… on the [U.S.-Mexico] border, I think asking for help is very hard.”*

Some participants described HPV and its vaccine as an issue primarily affecting females while concurrently underestimating the negative impacts on their own health.

“*…it's like to prevent cancer in women but [for] men, [HPV-related cancers] doesn't affect them…”*

Additionally, even when participants expressed knowledge about HPV and its vaccine, the historical framing of HPV as primarily a female health issue persisted:

“*I know HPV is a virus… I do know that there's a vaccine… linked to a lot of a different cancers, most notably cervical cancer… but I think like most men don't get [or like need] the vaccine, I think, but I don't know*.”

Participants also expressed discomfort with receiving health information from females, particularly on male health topics:

“*For me and most people, but for me, I would like that if someone is going to talk to me about men's issues... and it's not about being sexist because women are also very capable of giving this information, but at my age there's a lot of machismo that exists [in our culture] and when a woman tries to give [us] information, I've had cousins with that same attitude, but I see it like a man [would] so to try to give a man information…”*.

The final code, **stigma manifestations**, illustrates how stigma was experienced and expressed through language and attitudes that conveyed internalized fear, social discomfort, and perceived judgment in relation to HPV and its vaccine.

Participants described feelings of embarrassment, shame, and fear around disclosing or discussing HPV and sexual health, suggesting both perceived and anticipated stigma:

“*I don't know about HPV in general, but just with STDs, you know, people think like, oh, like once you have it, you have it forever. No matter what you do…”*

“*…with my [partners] to talk I guess about any sexual issues or to ask questions is kind of like frowned upon, it's not something anybody wants to talk about…”*

“*…like when you go in with a new partner and you're diagnosed, you have to be like: ‘oh, just to let you know, I have an STI', and for them it could be a make-or-break point…”*

Even in digital spaces designed to foster sexual connection, participants discussed their peer group's anticipated discomfort to discuss sexual health, to include HPV vaccination:

“*I think that [promoting the HPV vaccine on dating apps]… might make people avoid using them but I don't know because it's a taboo topic…”*

Participants also expressed frustration through experiences of secondary stigma, recounting how HPV-related health outcomes among females were dismissed within healthcare settings. One participant described how his family member's HPV concerns were minimized by providers, reinforcing mistrust in the healthcare system:

“*…so I'm like okay, you downplayed to the sense where you should be fine, and there's nothing for her to worry about… instead of them scheduling a checkup… they just played it off as if nothing had never happened…”*

Another participant reflected on possible internalized stigma, articulating a sex-specific stereotype that frames male sexuality as careless and reactive:

“*The male gender I would consider is more careless in that sense… there's ways to prevent and to be protected, and I guess, guys would rather take the morning after pill rather than wear a condom…”*

This perception of male carelessness was also tied to the belief that HPV poses fewer health risks to males than to females, reinforcing sex-specific assumptions about vulnerability:

“*So… one of the ways [HPV] can affect guys… is not as severe as [it is for] females… since it affects more one gender than the other, only because one gender is more careless in that sense.”*

Together, these narratives illustrate how internalized, perceived, and anticipated stigma operate through language and attitudes, reinforcing silence, discomfort, and avoidance of HPV-related discussions and vaccination.

#### 3.3.2 Interpersonal level

##### 3.3.2.1 Cultural and familial forces

This theme captures how discourses of intergenerational silence, religious morality, and sexual health taboos sustain health-related stigma and misinformation about HPV and vaccination at the interpersonal level. Participants articulated how limited parental communication about sexual health and inadequate formal education constrained open discussion about HPV and vaccination.

Multiple codes illustrated this theme. For example, the code **intergenerational transmission of cultural and familial silence around sexual health** highlights how Hispanic households, shaped by cultural norms, religious values, and taboos, suppressed open discussion of sexual health. This silence contributed to limited awareness, discomfort with sexual health conversations, and delays in seeking preventive care such as HPV vaccination:

“*Sex wasn't something that we spoke about very much…those are topics that we never touched…*”

Another participant recalled his father's silence and religious framing of sex reinforced shame and avoidance:

“*…when I was living with my father, he didn't like talking about sex and since religion was a big part of our lives, his [view] was that if we had sex, that it was against God and it was a sin, and we'd go to hell…”*

Participants also reflected on how such silences are transmitted across generations particularly within the U.S.–Mexico border context:

“*…if your parents don't speak with you about [sexual health, it's] because they weren't taught those things either when they were growing up then you don't learn about that…”*

The additional code, **misinformation and fear about vaccines**, illustrates how knowledge gaps, reproductive health myths, and broader skepticism about vaccines shaped male hesitancy toward HPV vaccination. These concerns were not expressed in isolation but were entangled with distrust toward other vaccines, such as those for COVID-19.

Participants expressed uncertainty when making vaccine decisions without adequate guidance:

“*I don't know if [it's my] lack of education about the topic, whether it's HPV or COVID, but there's something that raises doubts when making a [vaccine] decision…”*

Another participant reflected on his own apprehension and fear about both HPV and COVID-19 vaccines:

“*First of all, I didn't know that there was [an HPV] vaccine much less that it was 3 doses. I think that a lot of people are afraid; I'm scared of getting vaccinated with the COVID vaccine, I haven't gotten it, I'm simply afraid and I would say that I'm afraid of getting the HPV vaccine as well*.”

Participants also expressed fear and misunderstandings surrounding about vaccine technology, particularly regarding their development and potential risks:

“… *from what I understand, they're inventing something against [HPV] but since a lot of people are afraid of HPV infection, they might also have the same fear toward the vaccine as they do for the infection*.”

Myths about fertility and reproductive consequences further facilitated health-related stigma, as one participant explained:

“*I've also heard from several acquaintances who've been told that they might not have any problems [if they get vaccinated] but that if they have kids someday, they might have defects that their kids might come out with Down syndrome or something similar.”*

Participants also described a climate of social hesitation, where individuals delayed vaccination until others demonstrated that it was safe:

“… *people are like either anti-vaccine or extremely like ‘oh I want to see, like everyone take it before, I take it…”'*

Collectively, these accounts demonstrate how intergenerational norms and cultural taboos function as interpersonal processes and mechanisms of stigma, limiting sexual and reproductive health knowledge and perpetuating sex-specific inequities in HPV vaccination.

#### 3.3.3 Systemic level

##### 3.3.3.1 Structural barriers to vaccination

This theme reflects how policy environments, institutional practices, and health system norms perpetuate systemic-level obstacles to HPV vaccination. Participants articulated how conservative political resistance to comprehensive sex education, gaps in male-targeted health messaging, and economic constraints functioned as key deterrents to vaccine uptake.

This theme encompassed several related codes. For example, the code **institutional norms, policies, and decision-maker influence** highlights how sex-specific healthcare policies and conservative political leadership at the state level constrained comprehensive sexual and reproductive health education and limited the inclusivity of HPV prevention efforts.

Participants described how policy decision-making, shaped by conservative ideologies, reinforced the framing of HPV as a female issue and restricted access to comprehensive education and vaccination:

“*… [Texas male state representatives] are always against [HPV] vaccines… [even for] young girls [which] has a big effect because [they] decide the type of education we receive in our schools [and] who's gonna have access to these vaccines…”*

Another participant reflected on how this environment silenced discussions about HPV in schools:

“*I don't know if it's because we live in a more conservative state, but we never talked about this topic [of HPV] in school.”*

Participants further connected education policy directly to political leadership:

“*…the people who approve what needs to be taught are Conservatives; the majority of people who make decisions are conservative men who are against vaccines…”*

“*…the topic of HPV, I think that schools should have that kind of education to prepare kids… but I don't think they're doing it well*…”

One participant elaborated on the stagnation of policy progress in Texas:

“*Texas is a state that doesn't want to change and in regard to the topic of sex education, I don't see it changing…”*

The local context of El Paso was also noted:

“…*sex education here in El Paso schools isn't comprehensive like other parts of the U.S., [or] like in Austin [Texas]…”*

A related set of codes, **delayed or lack of vaccination** and **persistent sex-based health disparities**, illustrates the direct and measurable effects of health-related stigma on health behaviors, such as HPV vaccination. Participants described limited prior exposure to HPV vaccine information, prompts, or engagement from healthcare providers, reflecting a lack of guidance and confidence in making vaccination decisions:

“*… what is the typical age range [or] like approved [age] of who should be getting the HPV vaccine in males…”*

Despite the knowledge gap, participants demonstrated interest in learning more when prompted:

“*I think that more information should be given about the ages that men, specifically, can get [the vaccine] after 26 years old and what cancers that [HPV] can cause. I know about [HPV] more or less but I still have doubts, I still need answers like what else can happen to men and also about getting this vaccine.”*

Participants also highlighted how institutional norms within the U.S. healthcare system reinforce the delayed engagement of males in preventive care. One participant reflected on how routine checkups and continuity of care for adult males tend to occur later in life compared to females, limiting opportunities to build trust with providers and normalize preventive behaviors:

“*[In the U.S.]… routine checkups don't start until much later for [adult] males… if we had more routine checkups… or [if we] were [closer] with [our] doctor and didn't really have that sense of shame…”*

Additionally, participants also highlighted how male caregiving and financial responsibilities often took precedence over their own health needs, contributing to delayed or forgone preventive care, including HPV vaccination:

“*I mean, some people have it a little tougher only because, I mean, comes down to a choice ‘would you rather get the vaccine now, or would you rather have food on the table' so, I guess, in my predicament I'm not that well-off, I'm still trying to finish [my] degree as well as raising my daughter, so, I, I have a choice to make, whether I eat or she eats, or she is vaccinated and I'm not, and in my situation I would rather make sure she has everything and I suffer a little so she is well-off in the future, but not everybody sees it in that way.”*

These findings underscore how institutional and political discourses shape access to sexual and reproductive health education and care, reproducing systemic inequities and reinforcing male exclusion from HPV prevention efforts.

#### 3.3.4 Multi-level

##### 3.3.4.1 Stigma resistance

This final theme reflects counter-narratives that resist ‘stigma marking' across individual, interpersonal, and systemic levels. It was informed by the code **stigma resistance**, which captures participants' statements that challenged dominant norms and articulated alternative, affirming approaches to sexual and reproductive health, such as that of HPV vaccination. This code encompassed discursive strategies aimed at normalizing vaccination, reframing it as a preventive health behavior rather than a source of shame or taboo, and integrating it into broader cultural identity.

Participants described educational and cultural strategies to disrupt stigmatizing narratives, increase collective awareness, and embed vaccination into community norms:

“*[Make it] attractive to get the vaccine, remove the stigma of the whole STD thing? And just painted [it] as… a preventive measure… [not] like taboo.”*

“*…so I think just general Sex Ed would help, like if people knew that [HPV-related] diseases are out there, and that there are [vaccines] not 100 percent, but like 90 something percent effective against [them]…”*

“*And I think in the same way, like, the more that people get vaccinated, and it becomes part of our culture, and it becomes part of our like identity, like, then I think it might be different*.”

These accounts reveal how participants embraced their identities as sexual beings and leveraged collective responsibility to reframe vaccination as a normative, health-promoting practice, illustrating the potential of community-driven discourses to challenge stigma processes and mechanisms and foster male engagement in HPV prevention.

Collectively, these findings illuminate the multi-level determinants and intersecting stigmas (Figure 1) that shape adult males' HPV vaccine beliefs, behaviors, and access within the U.S.–Mexico border context. Participants' narratives highlighted how interpersonal silences, cultural and structural barriers, and entrenched sex-specific norms reinforce exclusion from HPV prevention efforts yet also revealed pathways of resistance through community-driven discourses and affirming approaches to sexual and reproductive health. These findings are situated within the broader literature on health-related stigma, masculinity, and HPV prevention, with consideration of their implications for research, policy, and practice aimed at fostering more inclusive engagement of males in sexual and reproductive health initiatives.

## 4 Discussion

### 4.1 Synthesis, summary, and interpretation

This study provides an in-depth examination that helps advance the understanding of adult male HPV vaccination engagement within a U.S.-Mexico community context by demonstrating how stigma processes and mechanisms—rooted in masculine norms, sexual health taboos, intergenerational silence, misinformation, and institutional exclusion—shape HPV vaccination access, beliefs, and behaviors. Guided by a critical constructivist paradigm, the analysis was grounded in the recognition that knowledge, attitudes, and behaviors surrounding HPV vaccination are socially and historically constructed through unequal power relations (31). By applying the Critical Discourse Analysis (CDA) approach and the Health Stigma and Discrimination Framework (HSDF), this study illuminated how stigma processes and mechanisms operate as discursive, relational, and systemic forces: constraining knowledge and agency, discouraging engagement in sexual and reproductive health discussions, and limiting opportunities for preventive care (20, 21, 45).

Male HPV vaccine hesitancy and delayed uptake may be better understood as outcomes of intersecting stigma processes operating across social-ecological levels rather than simply individual knowledge deficits or attitudinal resistance. By tracing how interrelated stigmas intersect and compound across individual, interpersonal, and systemic levels, our findings help to demonstrate how HPV vaccine engagement is shaped not only by personal beliefs or choices, but by culturally embedded narratives, institutional silences, and policy-level exclusions that collectively position males as peripheral to sexual and reproductive health. This aligns with intersectional and cross-cutting approaches to health-related stigma, emphasizing how overlapping social identities and structural inequalities shape unique experiences of stigma and healthcare access (21, 52). Stigma-related avoidance of STI and HPV vaccination aligns with prior research showing that health-related stigmas act as significant barriers to health-seeking behaviors (20, 21). Male HPV vaccine hesitancy and delayed uptake may be better understood as outcomes of intersecting stigma processes operating across social-ecological levels rather than simply individual knowledge deficits or attitudinal resistance.

A key contribution of this study lies in its discursive analysis of how HPV is constructed as a “female issue,” often expressed through rhetorical distancing and hedging language which supports earlier findings showing that despite rising HPV awareness among males, misconceptions about their own vaccine eligibility and vulnerability persist, even among college-educated males (23, 24, 53). In doing so, this study adds nuance to prior research documenting male HPV knowledge gaps by showing how such gaps are actively produced and maintained through discourse rather than merely reflecting informational absence.

Importantly, this analysis reframes masculine norms not as static individual traits, but as sociocultural norms which are reinforced through education, healthcare, and political systems that perpetuate health-related stigma and limit male engagement in care. These findings align with prior research documenting the cultural tension between dominant norms that exclude males from prevention messaging (21, 22, 24). These dominate masculine norms manifest as “masculine ideals” that position help- and health-seeking behaviors as deviations from dominant expectations. These dynamics intersect with regional inequalities that contribute to compounding stigmas among Hispanic males, within the U.S.–Mexico border context, which may exacerbate broader patterns of cancer prevention disparities (12, 13, 23, 27, 54). This is further supported by evidence revealing that foreign-born Hispanics are among the lowest HPV-vaccinated groups, and that racial and ethnic minority populations disproportionately experience cancer-related health disparities (28, 29, 55, 56).

Intergenerational silence and religiously-informed sexual moralism further amplify these dynamics by shaping early understandings of sexuality as taboo, dangerous, or shame-laden, leaving misinformation related to vaccines—particularly fears surrounding fertility and safety—to fill the discursive vacuum (20, 21). These findings echo research showing that health systems fail to meaningfully engage adult male patients not only through lack of tailored messaging but also through the absence of male-identifiable messengers and advocates (1, 9, 23, 24). This study may help further understand how stigma is socially reproduced not only through overt discrimination, but through gaps and absences in education, communication, and health promotion.

Despite structural and cultural barriers, however, participants also articulated counter-narratives that resisted stigma and reframed HPV vaccination as a proactive, responsible, and relational health behavior (24, 48). These resistance narratives challenged dominant discourses depicting males as careless and disengaged, instead highlighting their agency, insight, and willingness to engage in prevention when supported by inclusive, affirming systems (21, 24, 48). Participants called for culturally resonant, sex-positive education and messaging that validated their roles in prevention—perspectives largely absent from prior HPV prevention research. These findings highlight opportunities for interventions that align with adult male's lived experiences and capacities for resisting health-related stigma (21, 24, 48). By linking policy discourse to lived experience, this study may help to better inform existing critiques of HPV prevention approaches that continue to prioritize female-centered vs. age- and gender-inclusive models of care (9, 10, 24).

Collectively, these findings illustrate how health-related stigma is shaped by what is (un)said—through institutional silences, cultural taboos, and discursive patterns that marginalize males in HPV prevention (23, 24). The HSDF helped trace the key processes and mechanisms through which stigma operated across socioecological levels, while CDA illuminated the power dynamics embedded in culture, norms, and policy (21, 45). Our study reframes HPV vaccine hesitancy not as an individual failing but as a collective outcome of intersecting individual, cultural, and structural forces (21). Therefore, language and discourse emerge as both barriers and opportunities—mechanisms through which stigma is sustained but also spaces where it can be disrupted (20, 45).

### 4.2 Strengths and limitations

Strengths of this study include the integration of male perspectives into public health discourse, which remains underexplored in HPV research (24, 54). The group interview format promoted collective reflection, peer dynamics, and layered discussion that would have been less likely in one-on-one interviews (21). Male participants were not only narrators of their HPV vaccination experiences but also active agents in questioning stigma, expressing resistance, and naming systemic barriers related to health-promoting behaviors (21). The use of CDA enhanced the depth of interpretation by elucidating how power, ideology, and language shape both overt and subtle forms of stigma processes and mechanisms (45). Additionally, the framework-informed coding scheme follows best practices as it was developed iteratively and informed by a local vaccine-eligible male health researcher, providing a model for future qualitative work on male health-related stigma and vaccination (31, 32).

Nevertheless, limitations remain. The purposive sample was small and context-specific, which limits generalizability and transferability. Participants were recruited from a single region (El Paso), characterized by unique sociopolitical, cultural, and healthcare dynamics that may not reflect those of other ethnically or geographically similar populations. Additionally, more than half of our study participants reported obtaining at least a university degree and more than half received at least one dose of the HPV vaccine which may further limit generalizability. Due to this, those without a college education or without a history of HPV vaccination may have been underrepresented. Despite these limitations, however, findings from our study may help shed light on similar healthcare utilization barriers, such as stigma, that contribute to health disparities. While the group setting facilitated rich discussion, it also introduced the possibility of social desirability bias among participants. Furthermore the group setting may have also prevented participants from discussing, expressing, or disclosing their true attitudes, experiences, and beliefs related to the sensitive issues of sexual health and manhood (masculinity). Additionally, qualitative interpretation is inherently subjective; although reflexivity was built into the study design, the researchers' positionalities and perspectives likely influenced how the data were framed and understood. This potential for interpretive subjectivity is particularly relevant in cross-sex research contexts, where male behaviors and outcomes are interpreted by researchers who may not share the same identities or lived experiences. These dynamics highlight the value of ongoing reflexivity and diverse research perspectives to identify “blind spots” in power relations, as even community-embedded researchers may inadvertently under- or overlook inequities that are more visible to outsiders.

### 4.3 Implications

Due to the qualitative and highly contextual nature of the data, our implications have limited transferability but nevertheless may provide a better understanding of male health and healthcare-seeking behaviors. However, given that male perspectives have historically been underexplored in health promotion research, particularly those of young adult Hispanics living in a U.S.-Mexico border region; public health intervention strategies for HPV and other sexual and reproductive health concerns should address the masculine norms, cultural silence, misinformation, and exclusionary systemic practices that surround male health and healthcare-seeking behaviors. Interventions should center male narratives and promote male-informed messaging that challenge traditional masculine norms while affirming their roles in preventive health.

However, systemic reform is perhaps another area to further explore; the minimal or limited understanding of males' perspectives in HPV prevention efforts may reflect broader health policy-level resistance to comprehensive, inclusive sexual and reproductive health education. Structural stigma processes and mechanisms—manifested through conservative educational standards and the absence of sex-inclusive health messaging—could also be addressed as they may be reinforcing negative beliefs and behaviors in healthcare seeking and utilization.

Narratives from this study revealed how stigma processes and mechanisms coalesce to sustain low HPV vaccine engagement among males. Health promotion efforts that ignore these dynamics may risk reproducing the exclusions that they aim to address. Public health campaigns should harness the strengths expressed by the priority populations they aim to serve. Many participants voiced interest in protecting their partners and families and resisted sex-specific stereotypes that portray them as careless or disengaged. These motivations offer a foundation for strength-based, community-embedded approaches that include male perspectives and cultivate agency.

Public health efforts should also be cognizant of the deep-rooted dimensions of masculine norms and create discursive spaces where males are included to help inform prevention efforts. Health promotion strategies should include culturally resonant messaging that considers how content is framed, who delivers it, and whether it affirms or undermines male agency and identity. By fostering more inclusive and affirming health communication, public health programs can help normalize male engagement in HPV vaccination and broader preventive healthcare.

## 5 Conclusion

Our study examined how stigma processes and mechanisms—rooted in masculinity, cultural silence, institutional exclusion, and misinformation—shape HPV vaccination access, beliefs, and behaviors among vaccine-eligible adult males in a U.S.–Mexico border context. Through a critical constructivist lens, the findings revealed that these stigma processes and mechanisms operate discursively and structurally, marginalizing males from prevention efforts while also creating opportunities for resistance and agency.

By uncovering how stigma is constructed, reproduced, and contested across social-ecological levels, this study contributes to a more nuanced understanding of male health behaviors and highlights the importance of inclusive, equity-focused public health approaches. In doing so, it challenges prevailing narratives that frame males as disengaged, instead positioning them as capable of meaningful engagement when supported by affirming, culturally resonant systems.

## Data Availability

The datasets presented in this article are not readily publicly available due to participant confidentiality and institutional oversight requirements. Deidentified individual participant data (IPD) and supporting study materials may be made available on a case-by-case basis following a formal request to the University of Texas at El Paso (UTEP) Institutional Review Board (IRB) and approval by the Principal Investigator (PI) of the parent study. Requests to access these datasets should be directed to UTEP IRB, irb.orsp@utep.edu.
